# White Matter Hyperintensity Burden and Decline in Driving Performance Among Older Adults

**DOI:** 10.1001/jamanetworkopen.2025.54501

**Published:** 2026-01-29

**Authors:** Madhur Parihar, Yasheng Chen, Byron Xu, Semere Bekena, Ramkrishna K. Singh, Yiqi Zhu, Jean-Francois Trani, Jin-Moo Lee, David B. Carr, Chia-Ling Phuah, Ganesh M. Babulal

**Affiliations:** 1 Barrow Neuro Analytics Center, Barrow Neurological Institute, Phoenix, Arizona; 2 Department of Neurology, Washington University School of Medicine, St Louis, Missouri; 3 Institute of Public Health, Washington University in St Louis, St Louis, Missouri; 4 Department of Medicine, Washington University School of Medicine, St Louis, Missouri; 5 Department of Neurology, Barrow Neurological Institute, Phoenix, Arizona; 6 Department of Neurosurgery, Barrow Neurological Institute, Phoenix, Arizona; 7 Centre for Social Development in Africa, Faculty of Humanities, University of Johannesburg, Johannesburg, South Africa

## Abstract

**Importance:**

White matter hyperintensity (WMH) burden is associated with cognitive decline, but its association with driving performance among older adults and the associations of modifiable risk factors remain unclear.

**Objective:**

To evaluate the association of total and regional WMH burden with longitudinal naturalistic driving performance in cognitively normal older adults and evaluate moderating associations of cognitive decline and antihypertensive therapy.

**Design, Setting, and Participants:**

This prospective longitudinal cohort study enrolled community-dwelling older adults (≥65 years) in the Driving Real-World In-Vehicle Evaluation System Project. Baseline 3-T brain magnetic resonance imaging (MRI) scans and data from continuous in-vehicle driving monitoring were collected from January 1, 2015, to December 31, 2024 (mean [SD] follow-up, 5.6 [1.8] years), with annual cognitive and clinical assessments.

**Exposure:**

Total and regional WMH volumes, cognitive status change, and antihypertensive medication use.

**Main Outcomes and Measures:**

Monthly driving trip frequency, trip distance, unique destinations, driving entropy, and safety events. Longitudinal associations were evaluated using linear mixed-effects models adjusted for demographic characteristics, socioeconomic status, and vascular risk.

**Results:**

Among 220 participants (mean [SD] age, 72.9 [5.0] years; 119 men [54%]) with low vascular risk (mean [SD] Framingham Stroke Risk Profile, 6.4% [1.3%]), greater baseline WMH burden was correlated with lower driving frequency (β = −0.16; 95% CI, −0.27 to −0.06; *P* = .002), reduced driving entropy (β = −0.17; 95% CI, −0.27 to −0.06; *P* = .002), and fewer unique destinations (β = −0.17; 95% CI, −0.27 to −0.07; *P* = .001). Longitudinally, higher WMH was associated with faster declines in driving frequency (β = −0.08; 95% CI, −0.13 to −0.04; *P* < .001), entropy (β = −0.11; 95% CI, −0.17 to −0.06; *P* < .001), and unique destinations (β = −0.09; 95% CI, −0.14 to −0.04; *P* < .001). Growth in posterior WMH burden showed the strongest association with increased crash risk (β = 1.71; 95% CI, 1.17-2.24; *P* < .001) among those developing cognitive impairment (38 [17%]). Among participants, 135 used antihypertensive therapy and 113 underwent follow-up MRI. Antihypertensive therapy, particularly angiotensin-converting enzyme inhibitors, was associated with attenuated WMH-related risky driving before adjustment, but not after adjustment (β = −0.17; 95% CI, −0.37 to 0.03; unadjusted *P* = .02; false discovery rate–adjusted *P* = .08). Sensitivity analyses confirmed associations were independent of Alzheimer dementia pathology.

**Conclusions and Relevance:**

In this cohort study of older drivers, higher WMH burden, especially in posterior regions, was associated with progressive driving self-regulation and increased errors in those developing cognitive impairment. Antihypertensive use attenuated WMH-associated unsafe driving. Posterior WMH may represent a biomarker for identifying older drivers at risk and may inform mobility-preserving interventions in aging populations.

## Key Points

**Question:**

How is total or regional white matter hyperintensity (WMH) burden associated with longitudinal changes in naturalistic driving performance among older adults?

**Findings:**

In this cohort study of 220 cognitively normal older adults followed up longitudinally, greater baseline and progressive WMH burden, particularly in posterior regions, was associated with reduced driving frequency, complexity, and safety, especially among participants who later developed cognitive impairment. In the unadjusted model, antihypertensive therapy was associated with reduced WMH-related adverse driving behaviors; however, there was no association after adjustment.

**Meaning:**

This study suggests that posterior WMH burden may serve as a biomarker of increased driving risk in older adults.

## Teaser

This cohort study evaluates how white matter hyperintensity (WMH) burden is associated with longitudinal changes in naturalistic driving performance among cognitively normal older adults

## Introduction

White matter hyperintensities (WMHs) are well-established neuroimaging markers of cerebral small vessel disease^1,2^ and are associated with cognitive dysfunction and disability in older adults.^3,4,5,6^ However, the association of WMH with complex behaviors such as driving remains less defined.

Driving integrates cognitive, sensory, and motor functions,^7^ and subtle deficits can compromise safety and independence before overt cognitive decline emerges.^8^ Older adults with depression,^9,10^ preclinical Alzheimer dementia pathology,^11,12^ or cognitive impairment^13,14^ are at increased risk for driving errors and cessation, with early self-regulation of driving often signaling functional decline.^15,16^ However, the neurobiological basis of these changes and the role of WMH as a biomarker of declining driving performance remain unclear.

Emerging evidence links greater WMH burden with driving cessation,^17,18^ and severe leukoaraiosis with poorer driving performance and increased adverse outcomes.^19^ Most prior studies, however, are cross-sectional or self-reported,^18^ limiting insight into how WMH is associated with longitudinal driving trajectories in cognitively normal aging. New in-vehicle monitoring technologies now enable continuous, objective measurement of naturalistic driving,^20^ offering a unique window into early functional effects of brain aging.

The interplay among WMH, cognition, and vascular risk factors such as hypertension is also incompletely understood. Although antihypertensive medication use slows WMH progression^21,22^ and cognitive decline, its association with outcomes such as driving has not been systematically evaluated.

In this population-based cohort study of cognitively normal older adults, we used continuous driving data and serial neuroimaging metrics to examine associations of total and regional WMH burden with changes in driving performance. We also explored whether antihypertensive medication use modified these associations. Our findings aim to inform early identification of individuals at risk and strategies to preserve mobility and independence in aging.

## Methods

### Participants

Participants were community-dwelling older adults aged 65 years or older enrolled in the prospective Driving Real-World In-Vehicle Evaluation System (DRIVES) Project^23,24,25,26^ at Washington University School of Medicine, which examines aging and complex behaviors such as driving. Eligibility criteria included cognitive normalcy at baseline (Clinical Dementia Rating^27^ = 0 or Mini-Mental State Examination score ≥26) and a brain magnetic resonance imaging (MRI) scan within 2 years of starting longitudinal driving assessments. For analysis of longitudinal WMH change, we included 113 individuals with at least 1 follow-up MRI within 12 months after the baseline scan. All recruitment, written informed consent, and study protocols were approved by the Washington University School of Medicine institutional review board. This prospective cohort study followed the Strengthening the Reporting of Observational Studies in Epidemiology (STROBE) reporting guideline.

### Clinical, Cognitive, and Medication Data

Data for this study were collected from January 1, 2015, to December 31, 2024. Baseline sociodemographic information included self-reported racial and ethnic categories (African American or Black and White [the cohort comprised individuals of only these 2 races]), sex (male and female), years of education, and 5-digit zip codes. Data on race and ethnicity were collected as proxies for social determinants of health. The Area Deprivation Index, derived from zip code, served as a proxy for socioeconomic status. Participants underwent annual clinical and cognitive assessments, including Clinical Dementia Rating evaluations by experienced clinicians using semistructured interviews with participants and informants. Cognitive decline was defined as a Clinical Dementia Rating–Sum of Boxes score greater than 0.5 and/or Mini-Mental State Examination score less than 26 during follow-up. Common vascular conditions (myocardial infarction, diabetes, atrial fibrillation, and hypertension), vital signs (eg, systolic blood pressure), and self-reported medication data were collected annually following National Alzheimer Coordinating Center Uniform Data Set guidelines.^28^ A composite vascular risk score (modified Framingham Stroke Risk Profile [FSRP])^29^ was derived from clinical and medication data. Antihypertensive medication use was determined from detailed medication interviews at baseline and each annual follow-up visit. Medication initiation dates were verified to ensure therapy began before longitudinal driving monitoring. Medication classes are summarized in eTable 1 in Supplement 1.

### Naturalistic Driving Data

A commercial vehicle data logger (Azuga G2 tracking device; Aguza, a Bridgestone Co) was installed in the onboard diagnostic port of each participant’s vehicle to continuously record trip-level driving data. A trip was defined as the period from ignition start to off, with at least 100 m traveled. Collected data included time-stamped records of speed, latitude, longitude, and event-based threshold alerts for speeding, cornering, and hard braking. The DRIVES Project derived metrics such as total trips, mean distance traveled, number of unique destinations, trip distance distribution, speeding incidents, hard braking events, hard cornering (lateral acceleration >0.4*g*), driving entropy (route predictability), and radius of gyration (spatial stability).^24,30^ Driving data were processed and aggregated monthly for each participant, with values truncated at the 99th percentile to limit extreme values. Operational definitions for driving-related measures were as follows. Driving complexity reflected the variety and challenge of driving behavior, including number of unique destinations, driving entropy, and frequency of long-duration trips. Driving entropy quantified wayfinding or route variability, where higher values reflected less-routine navigation patterns. Driving errors referred to safety-critical events, including motor vehicle crashes (gravitational force, >2.5*g*, with recordings >20*g* excluded as likely sensor artifact [for context, airbags typically deploy at >20*g*]) and hard braking. Risky driving encompassed speeding or hard cornering events indicating aggressive or unsafe behavior.

### MRI Processing and WMH Assessments

Structural MRI was performed on 3-T scanners with T1-MPRAGE and 2-dimensional–fluid-attenuated inversion recovery (FLAIR) sequences. WMH volumes were derived using a validated convolutional neural network–based segmentation method.^31^ In brief, FLAIR and T1 images underwent standard preprocessing, including skull stripping, coregistration, bias field correction, and intensity harmonization, implemented with Statistical Parametric Mapping and FMRIB Software Library.^32^ Voxel-wise WMH probabilistic maps^31^ generated from FLAIR images were spatially normalized to the ICBM-152 template using Advanced Normalization Tools^33^ and FMRIB’s Linear Image Registration Tool.^32^ Voxels with a probability threshold of 50% or more were used to compute total WMH volume. Regional WMH estimates were obtained for 5 predefined spatial topographies^31^: juxtacortical, deep frontal, periventricular, parietal, and posterior. Total and regional WMH volumes were inverse-normal transformed prior to analysis.

### Positron Emission Tomography–Based Quantification of Alzheimer Dementia Pathology

Amyloid and tau positron emission tomography (PET) data within ±3 years of driving assessment initiation were included. Amyloid PET was performed a mean (SD) of 1.0 (0.9) years before, and tau PET 0.4 (1.8) years before, driving assessments. MRI-PET intervals averaged 0.04 to 0.4 years, indicating close temporal alignment. Amyloid burden (carbon 11–labeled Pittsburgh compound B or ^18^F-florbetapir) was expressed as centiloid values,^34^ and regional tau deposition (^18^F-flortaucipir) as standardized uptake value ratios.^35^ All scans underwent standardized Knight–Alzheimer Disease Research Center preprocessing and quality control.^36^

### Statistical Analysis

We examined associations between WMH and naturalistic driving performance using random coefficient linear mixed models. Driving metrics were modeled as a function of fixed effects: time, WMH (total or regional volumes), their interaction (time × WMH), and covariates (age at driving start, sex, race and ethnicity, years of education, Area Deprivation Index, and FSRP), with random intercepts and slopes (time | ID). WMH terms captured baseline associations, and time × WMH interactions tested longitudinal change. Monthly driving variables were assumed to vary linearly with WMH and covariates, all centered and scaled. Secondary models tested moderation by cognitive impairment, antihypertensive use (stratified by blood pressure control: poor control, >120/80 mm Hg from averaged serial measures), and medication class. Participants were classified as treated only if medication use overlapped 45% or more of their driving observation period. For longitudinal WMH analyses, we used change in relative regional WMH (WMH^region^/WMH^total^)follow-up − (WMH^region^/WMH^total^)baseline as the primary exposure variable to assess how change in spatial WMH distributions over time was associated with driving outcomes, restricted to driving data collected after each participant’s second MRI scan to maintain temporal ordering. Sensitivity analyses in the PET subgroup adjusted for centiloid and tau summary measures to examine WMH associations independent of Alzheimer dementia pathology. All analyses were conducted in R, version 4.4.2 using the lmerTest package (R Project for Statistical Computing). Marginal mean values for adjusted driving variables were derived using the R package ggeffects. To correct for multiple testing across 16 driving metrics and 5 WMH regions (80 total comparisons), false discovery rate adjustment was applied with significance set at a 2-sided false discovery rate–adjusted *P* < .05.

## Results

A total of 220 participants (mean [SD] age, 72.9 [5.0] years; 119 men [54%] and 101 women [46%]; 26 African American or Black [12%] and 194 non-Hispanic White [88%]) met inclusion criteria. Baseline demographic and clinical characteristics are presented in Table. Most participants were college educated and of average socioeconomic status. Vascular risk, based on the 10-year revised FSRP, was low (mean [SD] FSRP, 6.4% [1.3%]). The mean (SD) total WMH volume was 19.3 (12.7) cm^3^, with greater posterior than anterior lesion burden. Over a mean 5.6 (1.8) years of follow-up (range, 0.6-9.7 years), 38 participants (17%) developed cognitive impairment: 20 (53%) with Alzheimer dementia, 1 (3%) with vascular dementia, and 14 (37%) of uncertain etiology. Participants contributed a mean (SD) of 275.8 (92.9) weekly trip reports during follow-up. Amyloid and tau PET data were available for 112 participants (51%), with median intervals to driving assessment of 13.3 months (IQR, 4.7-20.2 months) prior and 6.5 months (IQR, 5.9-14.4 months) prior, respectively. Four participants (2%) were lost to follow-up, all with fewer than 2 years of data; 19 participants died but had complete data until death.

**Table.** Participant Baseline Demographic Characteristics (N = 220)

Characteristic	Mean (SD) [range]
Age, y	72.9 (5.0) [63.0-92.0]
Education, y	16.6 (2.2) [12.0-24.0]
Sex, No. (%)	
Female	101 (46)
Male	119 (54)
Race, No. (%)	
African American or Black	26 (12)
White, No. (%)	194 (88)
Antihypertensive medications, No. (%)	135 (61)
Area Deprivation Index	43.6 (22.4) [2.0-100.0]
WMH volume, cm^3^	
Total WMHs	19.3 (12.7) [2.4-71.0]
Juxtacortical WMHs	2.0 (2.8) [0.0-17.5]
Frontal WMHs	2.0 (2.5) [0.0-15.0]
Periventricular WMHs	3.9 (1.5) [0.7-7.3]
Parietal WMHs	5.0 (2.4) [0.5-10.8]
Posterior WMHs	6.5 (5.0) [0.7-25.9]
10-y Framingham Stroke Risk Profile, %	6.4 (1.3) [3.2-10.3]
Follow-up duration, y	5.6 (1.8) [0.6-9.7]

Abbreviation: WMHs, white matter hyperintensities.

### Association of WMH Burden With Greater Driving Self-Regulation

**Figure 1.**
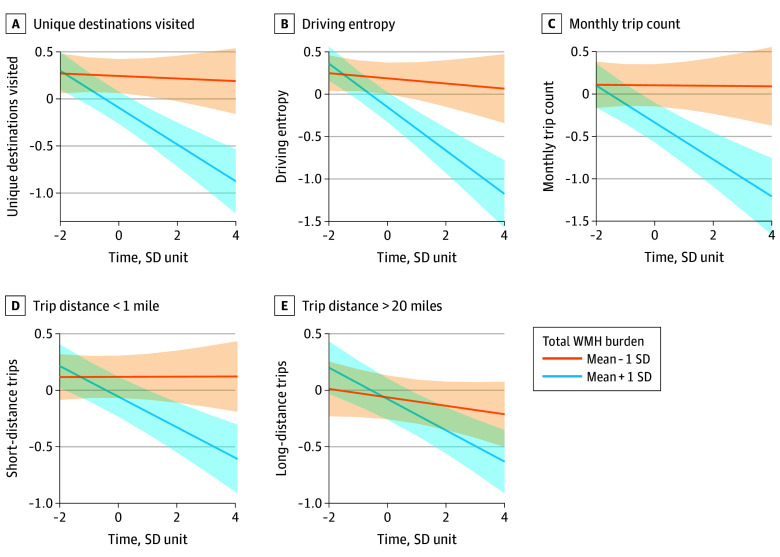
Total White Matter Hyperintensity (WMH) Burden and Longitudinal Change in Driving Frequency and Complexity Trajectories represent estimated marginal means of driving outcomes across standardized time, estimated from the fitted model and adjusted for covariates (age, sex, race, years of education, socioeconomic status, composite vascular risk, and individual variation in time slopes [random effects]), comparing participants with total WMH burden at mean − 1 SD (lower burden group) vs mean + 1 SD (higher burden group). Shaded areas indicate 95% CIs corresponding to each group.

Higher total WMH burden was significantly associated with reduced driving frequency and complexity. At baseline, greater WMH volume was associated with fewer overall trips (β = −0.16; SE = 0.05; 95% CI, −0.27 to −0.06; *P* = .002), fewer unique destinations (β = −0.17; SE = 0.05; 95% CI, −0.27 to −0.07; *P* = .001), and reduced driving entropy (β = −0.17; SE = 0.05; 95% CI, −0.27 to −0.06; *P* = .002) (Figure 1; eTable 2 in Supplement 1). Over time, higher total WMH burden was associated with steeper declines in monthly trips (β = −0.08; SE = 0.02; 95% CI, −0.13 to −0.04; *P* < .001), including both very short (<1 mile [<1.6 km]; β = −0.07; SE = 0.02; 95% CI, –0.11 to –0.03; *P* < .001) and very long (>20 miles [>32 km]; β = −0.05; SE = 0.02; 95% CI, –0.09 to –0.01; *P* = .02) distances, as well as greater reductions in unique destinations (β = −0.09; SE = 0.02; 95% CI, −0.14 to −0.04; *P* < .001) and driving entropy (β = −0.11; SE = 0.03; 95% CI, −0.17 to −0.06; *P* < .001) (Figure 1; eTable 2 in Supplement 1).

### Association of Regional WMH Burden With Distinct Driving Patterns

**Figure 2.**
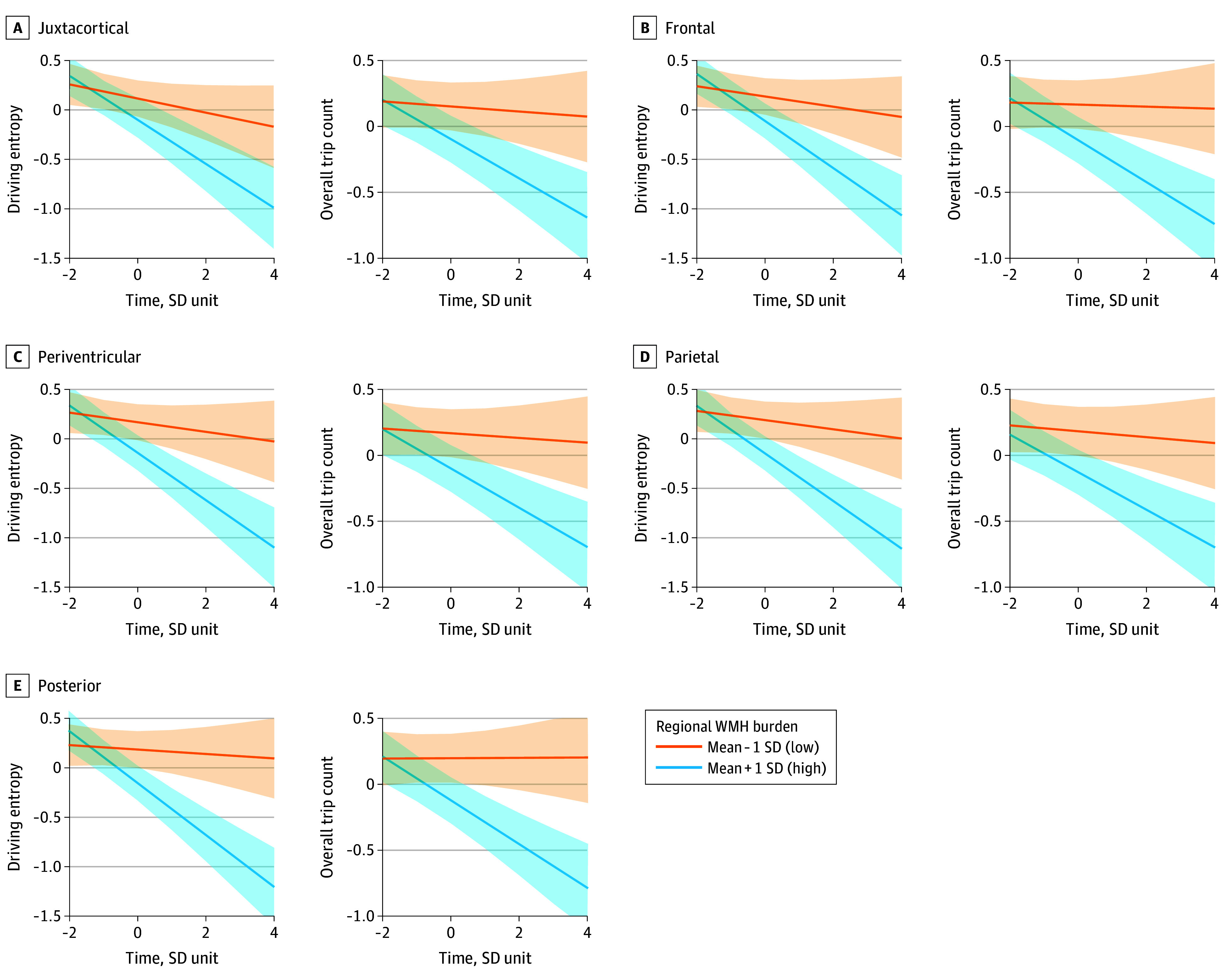
Region-Specific White Matter Hyperintensity (WMH) Burden and Longitudinal Change in Driving Frequency and Complexity Trajectories represent estimated marginal means of driving outcomes across standardized time, estimated from the fitted model and adjusted for covariates, comparing participants with regional WMH burden at mean + 1 SD (higher burden group) vs mean − 1 SD (lower burden group) for 5 white matter regions: (A) juxtacortical, (B) frontal, (C) periventricular, (D) parietal, and (E) posterior. Shaded areas indicate 95% CIs for each group.

Regional analyses revealed an anterior-posterior gradient between WMH burden and driving behaviors. Greater posterior and parietal WMH burden was most strongly associated with fewer trips (posterior: β = −0.16; SE = 0.05; 95% CI, –0.26 to –0.06; *P* = .006; parietal: β = −0.16; SE = 0.05; 95% CI, –0.26 to –0.06; *P* = .006), lower driving entropy (posterior: β = −0.17; SE = 0.05; 95% CI, –0.27 to –0.06; *P* = .004; parietal: β = −0.17; SE = 0.05; 95% CI, –0.27 to –0.07; *P* = .004), and fewer unique destinations (posterior: β = −0.16; SE = 0.05; 95% CI, –0.26 to –0.06; *P* = .004; parietal: β = −0.17; SE = 0.05; 95% CI, –0.27 to –0.08; *P* = .003), relative to anterior regions (eTable 2 in Supplement 1). Posterior WMH thus appeared to have the greatest association with driving frequency and complexity among older adults. Longitudinally, higher posterior WMH burden was associated with the steepest declines in unique destinations (β = −0.10; SE = 0.02; 95% CI, –0.15 to –0.05 *P* < .001), driving entropy (β = −0.12; SE = 0.03; 95% CI, –0.18 to –0.07; *P* < .001), and overall driving frequency (β = −0.08; SE = 0.02; 95% CI, –0.13 to –0.04; *P* = .002). Representative trajectories of driving outcomes by regional WMH burden are shown in Figure 2.

### Associations Between WMH and Driving in Cognitively Impaired Individuals

**Figure 3.**
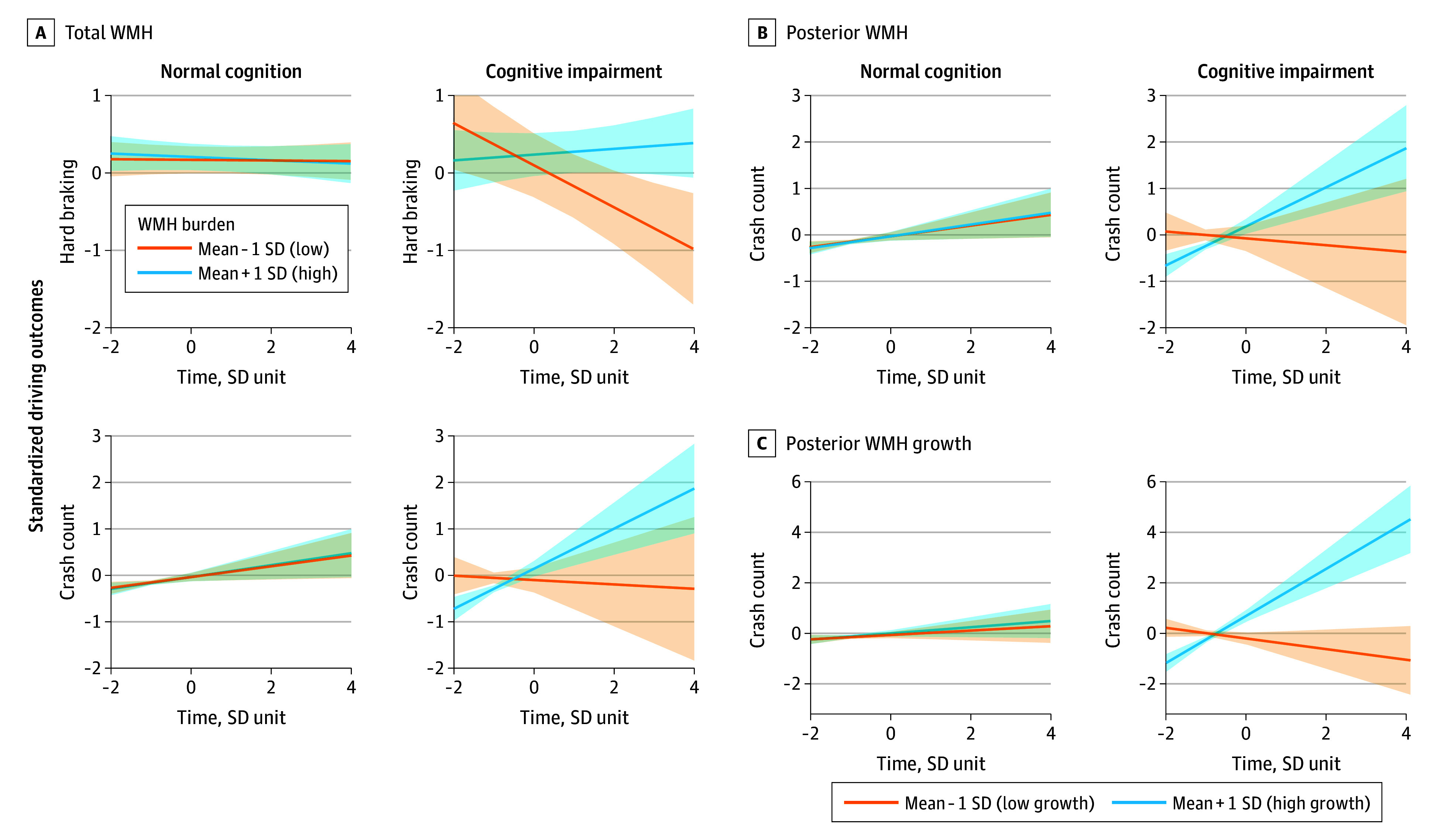
Longitudinal Changes in Driving Outcomes by White Matter Hyperintensity (WMH) Burden and Cognitive Status Trajectories represent estimated marginal means of standardized driving outcomes over standardized time, estimated from the fitted model and adjusted for covariates, comparing participants with high (mean + 1 SD) vs low (mean − 1 SD) total and posterior WMH burden, stratified by cognitive status (remained cognitively normal vs developed cognitive impairment). Shaded areas indicate 95% CIs for each group. Marginal effects plots show (A) the modeled 3-way interaction among time, total WMH burden, and cognitive status on hard-braking rates and crash counts; (B) the modeled association of posterior WMH burden and cognitive status with crash counts over time; and (C) the modeled association of posterior WMH burden growth and cognitive status with crash counts over time.

Among the 220 participants, 38 (17%) developed cognitive impairment during follow-up. They were older at baseline (mean [SD] age, 75.6 [4.2] vs 72.3 [5.0] years; *P* < .001), had greater total and regional WMH burden, were less likely to use antihypertensive medications, and had higher driving cessation rates (15 of 38 [44.1%] vs 30 of 182 [19.7%]; *P* = .006) (eTable 3 in Supplement 1). Cognitive status modified the longitudinal association between WMH burden and driving behavior. In participants who developed cognitive impairment, higher total WMH burden was more strongly associated with increases in driving errors over time, including higher crash rates (β = 0.23; SE = 0.11; 95% CI, 0.02-0.45; *P* = .03) and hard-braking events (β = 0.16; SE = 0.06; 95% CI, 0.05-0.27; *P* = .005), compared with cognitively normal participants (Figure 3). These individuals showed pronounced increases in crash rates (from <2 to 15-20 per year) and hard-braking events (from 3 to 7-8 per year) after year 6, diverging from cognitively normal participants whose rates remained low (eFigure in Supplement 1). Regionally, posterior WMH had the strongest association with driving errors, with an association for increased hard-braking events (β = 0.15; SE = 0.06; 95% CI, 0.04-0.26; *P* = .02) (eTable 4 in Supplement 1). In the unadjusted model, there was an association between posterior WMH and crash counts (β = 0.24; SE = 0.11; 95% CI, 0.03-0.45; unadjusted *P* = .02).

### Association of Posterior WMH Growth With Increasing Driving Risk

In 113 participants with longitudinal MRI (≥12 months postbaseline scan), posterior WMH growth was associated with declining driving performance. Greater posterior WMH expansion was correlated with higher crash counts (β = 0.43; SE = 0.15; 95% CI, 0.14-0.73; *P* = .02), with this association persisting over time (time × posterior WMH: β = 0.34; SE = 0.13; 95% CI, 0.08-0.61; *P* = .046). Participants with follow-up MRI had similar clinical and demographic profiles but lower baseline total and regional WMH burden (eTable 5 in Supplement 1). Secondary analyses showed the strongest association between posterior WMH progression and crash risk among individuals who subsequently developed cognitive impairment (β = 1.71; SE = 0.27; 95% CI, 1.17-2.24; *P* < .001), suggesting posterior WMH growth may serve as an early biomarker of declining driving safety and impending cognitive decline (Figure 3).

In sensitivity analyses adjusting for amyloid and tau PET biomarkers, greater posterior WMH burden remained associated with increased driving errors among individuals who developed cognitive impairment (crashes: β = 0.42; SE = 0.18; 95% CI, 0.07-0.77; *P* = .03; hard braking: β = 0.17; SE = 0.07; 95% CI, 0.03-0.31; *P* = .03) (eTable 6 in Supplement 1). Posterior WMH progression was also associated with higher crash risk longitudinally (β = 0.65; SE = 0.23; 95% CI, 0.19-1.12; *P* = .03), with significant association over time (β = 0.57; SE = 0.21; 95% CI, 0.15-1.00; *P* = .04).

### Antihypertensive Use and WMH-Related Risky Driving

At baseline, 135 participants (61%) reported taking antihypertensive medication, yet 114 of these individuals (84%) still had poorly controlled blood pressure (eTable 7 in Supplement 1). Compared with those not receiving antihypertensive therapy, medication users were less likely to develop cognitive impairment during follow-up (13% vs 28%; *P* = .008) and had higher mean (SD) FSRP (6.7 [1.2] vs 5.9 [1.5]; *P* < .001). Baseline WMH burden and duration of follow-up were similar across groups.

**Figure 4.**
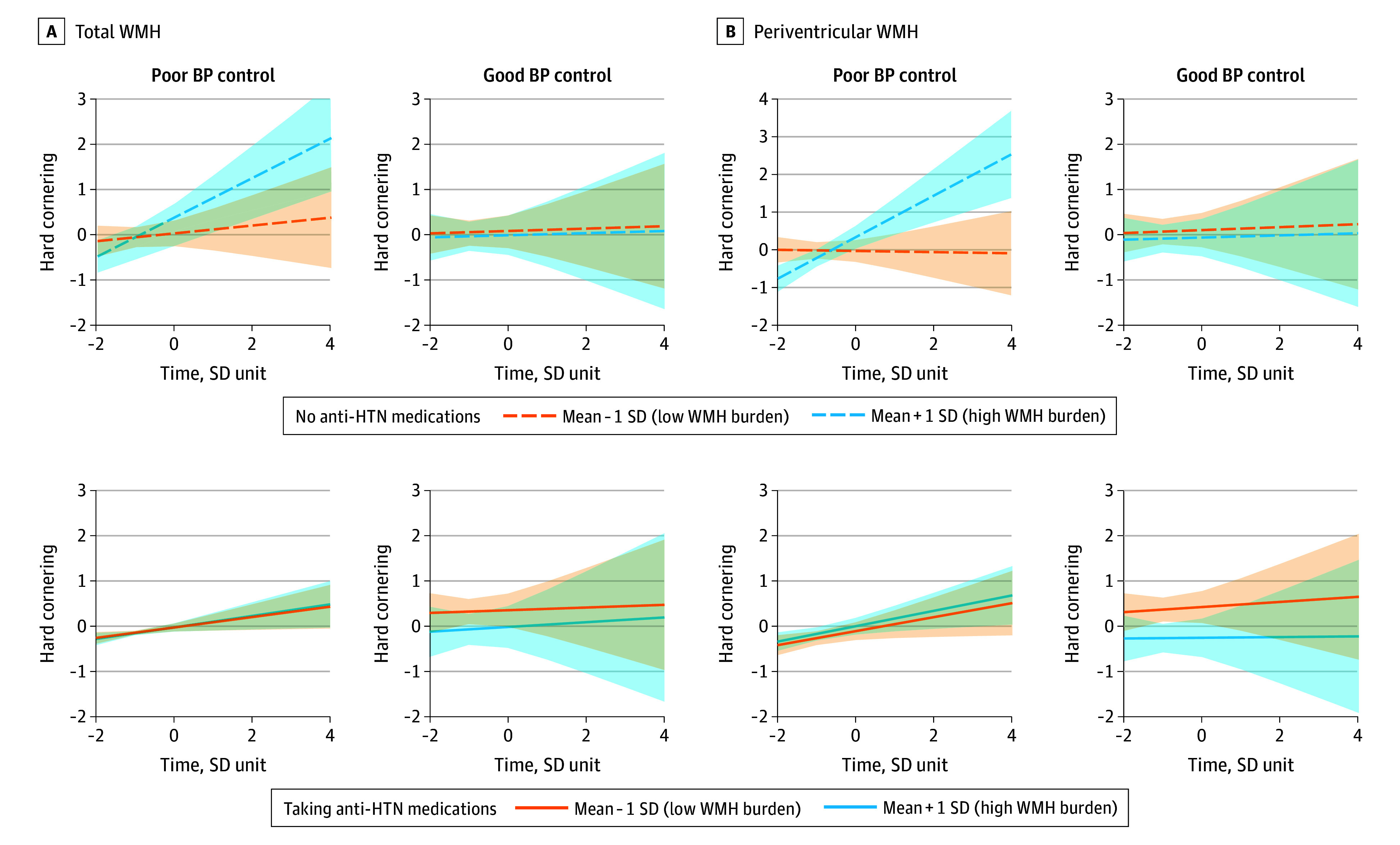
Interaction of White Matter Hyperintensity (WMH) Burden, Hypertension (HTN) Treatment, and Blood Pressure (BP) Control With Risky Driving Behavior Trajectories represent estimated marginal means of standardized driving outcome (rate of hard cornering) over standardized time, estimated from the fitted model and adjusted for covariates, comparing participants with high (mean + 1 SD) vs low (mean − 1 SD) (A) total WMH and (B) periventricular WMH burden, further stratified by HTN medication use and BP control status. Shaded areas indicate 95% CIs for each group.

Antihypertensive medication use modified the association of WMH burden with driving performance. Higher WMH burden was associated with increased hard cornering (β = 0.17; SE = 0.08; 95% CI, 0.01-0.34; *P* = .04), a marker of risky driving. Among participants with uncontrolled blood pressure, there was a significant 3-way interaction between time, periventricular WMH, and antihypertensive use. Antihypertensive use was associated with reduced risky driving in this group (β = −0.26; SE = 0.10; 95% CI, –0.44 to –0.07; *P* = .04) (eTable 8 in Supplement 1). Similar, but nonsignificant, trends were observed in participants with controlled blood pressure, regardless of medication status (Figure 4). Collectively, these findings suggest antihypertensive medications may be associated with neurovascular protection against WMH-related adverse driving, beyond the effects of blood pressure control alone.

Stratified analyses showed that the protective association of antihypertensive medication with WMH-related risky driving was specific to angiotensin-converting enzyme (ACE) inhibitors. Among individuals with poorly controlled blood pressure taking ACE inhibitors, WMH burden was associated with reduced hard cornering before adjustment, but there was no association after adjustment (β = −0.22; SE = 0.13; 95% CI, –0.48 to 0.03; *P* = .08; unadjusted *P* = .02). The largest reduction in hard cornering was observed for periventricular WMH before adjustment, but the association was not significant after adjustment (β = −0.31; SE = 0.13; 95% CI, –0.57 to –0.06; *P* = .08; unadjusted *P* = .02) (Figure 4; eTable 8 in Supplement 1). No significant moderating effects were observed for other antihypertensive classes, such as β-blockers, angiotensin receptor blockers, diuretics, or calcium channel blockers, highlighting a potential unique benefit of ACE inhibitors on WMH-related adverse driving.

## Discussion

Greater WMH burden, particularly in posterior regions, was associated with significant declines in driving behavior over time among community-dwelling, cognitively normal older adults. Using continuous, naturalistic driving assessments, we found that higher WMH burden was associated with decreased driving frequency, reduced driving complexity, and increased rates of adverse driving outcomes, including crashes. These findings underscore the clinical utility of in-vehicle monitoring to detect subtle cognitive and functional changes associated with cerebral small vessel disease.

Driving requires the dynamic integration of cognitive, sensory, and motor functions in response to external stimuli. Our findings extend prior evidence linking WMH with cognitive decline^1,3,4^ and behavioral adaptations such as increased driving self-regulation and cessation.^17,18,19^ Participants with higher WMH burden made fewer trips, visited fewer unique destinations, and demonstrated lower driving pattern entropy, reflecting a shift toward more cautious and restricted driving. These changes were evident for both short and long trips and became more pronounced over time, suggesting WMH accumulation may be associated with accelerated age-related reductions in driving activity. These driving adaptations likely reflect awareness and intentional self-regulation; preserved insight may underlie these adjustments, offering an indication that older adults with greater WMH burden can recognize limitations and actively manage their driving risk through appropriate behavioral modification.

Regional analyses revealed a clear anterior-posterior gradient, with parietal and posterior WMH exerting the strongest association with driving complexity and frequency. This finding supports prior evidence that posterior white matter tracts are critical for visuospatial and executive functions involved in safe driving.^37,38,39^ Clinically, progression of posterior WMH may represent an early marker of declining driving safety and future cognitive impairment.^3,37,40^ Longitudinally, higher posterior WMH burden was associated with the greatest declines in unique destinations visited, driving entropy, and frequency, highlighting the disproportionate association of posterior WMH with complex and adaptive driving behaviors.

Cognitive impairment further amplified associations between WMH burden and adverse driving outcomes. In participants who developed cognitive decline, higher WMH burden was associated with greater increases in driving errors, suggesting that WMH-related neural disruption may be associated with accelerated functional decline as cognitive reserve wanes.^3^ The association of posterior WMH with driving was independent of amyloid and tau pathology, consistent with additive associations rather than synergistic associations with Alzheimer disease biomarkers.

In exploratory analyses, antihypertensive medication use—particularly ACE inhibitors—was associated with attenuated associations between WMH burden and unsafe driving outcomes, especially in individuals with poorly controlled blood pressure. This protective association was not observed with other antihypertensive classes, consistent with findings from SPRINT-MIND (Systolic Blood Pressure Intervention Trial–Memory and Cognition in Decreased Hypertension) that ACE inhibitors uniquely mitigate WMH progression independently of blood pressure control.^41^ Such findings align with prior research suggesting some antihypertensives may exert neurovascular protective effects beyond blood pressure regulation, possibly through slowing cerebral small vessel disease progression or enhancing cerebral perfusion,^21,22,42,43^ or they may reflect differences in blood-brain barrier permeability or anti-inflammatory properties among antihypertensive agents.^44,45^

These findings highlight the value of vascular brain health surveillance in older adults, particularly through WMH burden screening among those with vascular risk factors or early cognitive symptoms. Our results support a shift toward earlier and more personalized approaches to preserving mobility and independence in aging populations. Targeted screening of vulnerable groups is increasingly feasible, with posterior WMH progression emerging as a promising biomarker for identifying individuals at risk for unsafe driving and functional decline. As sensor and passive vehicle monitoring technologies become more widespread, there is growing potential for unobtrusive risk stratification and digital health interventions to help maintain safe mobility for older adults.

### Strengths and Limitations

This study has some strengths, including a well-characterized cohort, robust driving data over 9 years, and serial neuroimaging with regional WMH quantification. The study also has some limitations. Generalizability is limited by the predominantly non-Hispanic White, college-educated sample. Medication use was self-reported and collected at baseline and annually, resulting in sparse data and possible misclassification. The 45% overlap threshold for medication adherence was a pragmatic choice given intermittent data but may add further misclassification. Dosage information was unavailable, precluding dose-response analysis. Residual confounding may persist despite adjustment for multiple key covariates. The observational, single-cohort design and limited sample size constrain causal inference and the detection of effects for less-common drug classes. These exploratory findings require replication in future studies.

## Conclusions

In this cohort study of older drivers, progression of posterior WMH burden emerged as a clinically meaningful biomarker for declining driving safety, especially among those at greater risk for cognitive impairment. The observed association between antihypertensive therapy and reduced WMH-related functional decline underscores the need for further investigation of targeted interventions and may inform future strategies to preserve mobility and independence in aging populations.

## Notes

Supplement 1. **eTable 1.** Antihypertensive Medications Included in Analysis **eTable 2.** Associations Between Total and Regional WMH Burden and Driving Behavior Outcomes **eTable 3.** Demographic, Clinical, and WMH Characteristics by Cognitive Status During Follow-Up **eFigure.** Longitudinal Change in Adverse Driving Events by Cognitive Status **eTable 4.** Main Effects of Total and Regional WMH Burden, Time, and Cognitive Impairment on Driving Outcomes **eTable 5.** Demographic, Clinical, and WMH Characteristics by MRI Follow-up Status **eTable 6.** Main Effects of Posterior WMH Burden, Growth, and Cognitive Impairment on Driving Safety Outcomes, With and Without AD Pathology Adjustment **eTable 7.** Demographic, Clinical, and WMH Characteristics by Antihypertensive Therapy Status **eTable 8.** Longitudinal Association Between Periventricular WMH and Driving Behaviors Varies by HTN Treatment and BP Control Status

Supplement 2. **Nonauthor Collaborators.** Driving Real-World In-Vehicle Evaluation System (DRIVES) Project

Supplement 3.
Data Sharing Statement
