# Supplementary material for: White Matter Hyperintensity Burden and Decline in Driving Performance Among Older Adults
Source: JAMA Netw Open. 2026 Jan 29;9(1):e2554501. doi: 10.1001/jamanetworkopen.2025.54501 (PMC12856682; doi:10.1001/jamanetworkopen.2025.54501)
Supplement: Supplement 1. — eTable 1. Antihypertensive Medications Included in Analysis eTable 2. Associations Between Total and Regional WMH Burden and Driving Behavior Outcomes eTable 3. Demographic, Clinical, and WMH Characteristics by Cognitive Status During Follow-Up eFigure. Longitudinal Change in Adverse Driving Events by Cognitive Status eTable 4. Main Effects of Total and Regional WMH Burden, Time, and Cognitive Impairment on Driving Outcomes eTable 5. Demographic, Clinical, and WMH Characteristics by MRI Follow-up Status eTable 6. Main Effects of Posterior WMH Burden, Growth, and Cognitive Impairment on Driving Safety Outcomes, With and Without AD Pathology Adjustment eTable 7. Demographic, Clinical, and WMH Characteristics by Antihypertensive Therapy Status eTable 8. Longitudinal Association Between Periventricular WMH and Driving Behaviors Varies by HTN Treatment and BP Control Status [file jamanetwopen-e2554501-s001.pdf]

Supplementary Online Content

Parihar M, Chen Y, Xu B, et al; Driving Real-World In-Vehicle Evaluation System (DRIVES) Project. White matter hyperintensity burden and decline in driving performance among older adults. *JAMA Netw Open.* 2026;9(1):e2554501. doi:10.1001/jamanetworkopen.2025.54501

eTable 1. Antihypertensive Medications Included in Analysis

eTable 2. Associations Between Total and Regional WMH Burden and Driving Behavior Outcomes

eTable 3. Demographic, Clinical, and WMH Characteristics by Cognitive Status During Follow-Up

eFigure. Longitudinal Change in Adverse Driving Events by Cognitive Status

eTable 4. Main Effects of Total and Regional WMH Burden, Time, and Cognitive Impairment on Driving Outcomes

eTable 5. Demographic, Clinical, and WMH Characteristics by MRI Follow-up Status

eTable 6. Main Effects of Posterior WMH Burden, Growth, and Cognitive Impairment on Driving Safety Outcomes, With and Without AD Pathology Adjustment

eTable 7. Demographic, Clinical, and WMH Characteristics by Antihypertensive Therapy Status

eTable 8. Longitudinal Association Between Periventricular WMH and Driving Behaviors Varies by HTN Treatment and BP Control Status

This supplementary material has been provided by the authors to give readers additional information about their work.

eTable 1. Antihypertensive Medications Included in Analysis

NACC drug ID	Name	Class
D00730	benazepril (Lotensin)	ACE Inhibitors
D00013	enalapril (Vasotec)	ACE Inhibitors
D00732	lisinopril (Prinivil, Zestril)	ACE Inhibitors
D03821	losartan (Cozaar)	Angiotensin Receptor Blockers (ARBs)
D04113	valsartan (Diovan)	Angiotensin Receptor Blockers (ARBs)
D00004	atenolol (Senormin, Tenormin)	Beta Blockers
D03847	carvedilol (Coreg, Carvedilol)	Beta Blockers
D00134	metoprolol (Lopressor, Toprol-XL)	Beta Blockers
D00689	amlodipine (Norvasc)	Calcium Channel Blockers (CCBs)
D00045	diltiazem (Cardizem, Tiazac)	Calcium Channel Blockers (CCBs)
D00051	nifedipine (Adalat, Procardia)	Calcium Channel Blockers (CCBs)
D00070	furosemide (Lasix)	Diuretics
D00253	hydrochlorothiazide (Esidrix, Hydrodiuril)	Diuretics
D03052	hydrochlorothiazide-triamterene (Dyazide)	Diuretics

© 2026 Parihar M et al. *JAMA Network Open.*

eTable 2. Associations Between Total and Regional WMH Burden and Driving Behavior Outcomes

	**Total WMH**			**Time*Total WMH**			**Posterior WMH**			**Time*Posterior WMH**		
	β	SE	*P* value	*β*	SE	*P* value	*β*	SE	*P* value	*β*	SE	*P* value
Total trips	**–0.16**	**0.05**	**0.002***	**–0.08**	**0.02**	**6e–04***	**–0.16**	**0.05**	**0.006***	**–0.08**	**0.02**	**0.002***
Trips <1 mile	–0.09	0.05	0.092	**–0.07**	**0.02**	**7e–04***	–0.09	0.05	0.179	**–0.07**	**0.02**	**0.003***
Trips 1 to 5 miles	–0.11	0.06	0.057	**–0.05**	**0.02**	**0.020***	–0.11	0.06	0.154	–0.06	0.02	0.065
Trips 5 to 10 miles	**–0.17**	**0.05**	**0.001***	–0.03	0.02	0.156	**–0.17**	**0.05**	**0.006***	–0.03	0.02	0.237
Trips 10 to 20 miles	**–0.10**	**0.05**	**0.047***	–0.03	0.02	0.277	–0.09	0.05	0.078	–0.03	0.02	0.559
Trips >20 miles	–0.01	0.05	0.886	**–0.05**	**0.02**	**0.021***	–0.01	0.05	0.970	–0.05	0.02	0.084
Unique destinations	**–0.17**	**0.05**	**0.001***	**–0.09**	**0.02**	**3e–04***	**–0.16**	**0.05**	**0.004***	**–0.10**	**0.02**	**6e–04***
Radius of gyration	0.00	0.02	0.941	0.00	0.02	0.877	0.00	0.02	0.996	0.01	0.02	0.850
Driving Entropy	**–0.17**	**0.05**	**0.002***	**–0.11**	**0.03**	**1e–04***	**–0.17**	**0.05**	**0.004***	**–0.12**	**0.03**	**2e–04***
Over-speeding	0.05	0.04	0.233	–0.04	0.02	0.057	0.05	0.04	0.437	–0.04	0.02	0.105
Crash count	0.02	0.03	0.443	0.04	0.03	0.207	0.02	0.03	0.480	0.05	0.03	0.242
Hard braking rates	0.02	0.05	0.620	0.01	0.02	0.720	0.02	0.05	0.947	0.01	0.02	0.967
Hard cornering rate	0.04	0.05	0.379	0.04	0.04	0.325	0.04	0.05	0.766	0.04	0.04	0.719
	**Parietal WMH**			**Time*Parietal WMH**			**Periventricular WMH**			**Time*Periventricular WMH**		
	β	SE	*P* value	*β*	SE	*P* value	*β*	SE	*P* value	*β*	SE	*P* value
Total trips	**–0.16**	**0.05**	**0.006***	**–0.06**	**0.02**	**0.011***	**–0.13**	**0.05**	**0.012***	**–0.07**	**0.02**	**0.008***
Trips <1 mile	–0.09	0.05	0.179	**–0.05**	**0.02**	**0.012***	–0.07	0.05	0.188	**–0.05**	**0.02**	**0.012***
Trips 1 to 5 miles	–0.13	0.06	0.143	–0.04	0.02	0.086	–0.09	0.06	0.175	–0.05	0.02	0.071
Trips 5 to 10 miles	**–0.16**	**0.05**	**0.006***	–0.01	0.02	0.777	**–0.12**	**0.05**	**0.016***	–0.03	0.02	0.242
Trips 10 to 20 miles	–0.04	0.05	0.389	–0.03	0.02	0.559	–0.09	0.05	0.078	–0.02	0.02	0.583
Trips >20 miles	0.00	0.05	0.970	–0.04	0.02	0.117	–0.02	0.05	0.970	–0.03	0.02	0.218
Unique destinations	**–0.17**	**0.05**	**0.003***	**–0.07**	**0.03**	**0.006***	**–0.13**	**0.05**	**0.013***	**–0.07**	**0.02**	**0.006***
Radius of gyration	–0.01	0.02	0.996	0.02	0.02	0.850	0.00	0.02	0.996	–0.02	0.02	0.850
Driving Entropy	**–0.17**	**0.05**	**0.004***	**–0.10**	**0.03**	**0.002***	**–0.15**	**0.05**	**0.005***	**–0.10**	**0.03**	**0.002***
Over-speeding	0.04	0.04	0.437	–0.02	0.02	0.282	0.01	0.04	0.746	–0.02	0.02	0.318
Crash count	0.02	0.03	0.480	0.05	0.04	0.242	0.03	0.03	0.480	0.05	0.03	0.242

© 2026 Parihar M et al. *JAMA Network Open.*

Hard braking rates	–0.01	0.05	0.947	0.02	0.02	0.967	0.00	0.05	0.947	0.00	0.02	0.967
Hard cornering rate	0.04	0.05	0.766	0.03	0.04	0.719	0.02	0.05	0.766	0.06	0.04	0.429
	**Frontal WMH**			**Time*Frontal WMH**			**Juxtacortical WMH**			**Time*Juxtacortical WMH**		
	β	SE	*P* value	*β*	SE	*P* value	*β*	SE	*P* value	*β*	SE	*P* value
Total trips	**–0.13**	**0.05**	**0.012***	**–0.08**	**0.02**	**0.003***	**–0.12**	**0.05**	**0.016***	**–0.07**	**0.02**	**0.008***
Trips <1 mile	–0.07	0.05	0.179	**–0.06**	**0.02**	**0.009***	–0.08	0.05	0.179	**–0.05**	**0.02**	**0.012***
Trips 1 to 5 miles	–0.07	0.06	0.277	–0.05	0.02	0.071	–0.06	0.06	0.304	–0.04	0.02	0.112
Trips 5 to 10 miles	**–0.14**	**0.05**	**0.008***	–0.05	0.02	0.050	**–0.14**	**0.05**	**0.009***	–0.06	0.02	0.050
Trips 10 to 20 miles	**–0.12**	**0.05**	**0.039***	–0.02	0.02	0.583	**–0.11**	**0.05**	**0.039***	–0.01	0.02	0.813
Trips >20 miles	0.00	0.05	0.970	–0.04	0.02	0.117	0.01	0.05	0.970	–0.03	0.02	0.228
Unique destinations	**–0.13**	**0.05**	**0.014***	**–0.09**	**0.02**	**0.001***	**–0.12**	**0.05**	**0.017***	**–0.07**	**0.02**	**0.006***
Radius of gyration	0.00	0.02	0.996	0.00	0.02	0.850	–0.01	0.02	0.996	0.01	0.02	0.850
Driving Entropy	**–0.12**	**0.05**	**0.022***	**–0.09**	**0.03**	**0.002***	**–0.11**	**0.05**	**0.039***	**–0.08**	**0.03**	**0.011***
Over-speeding	0.04	0.04	0.437	–0.04	0.02	0.105	0.06	0.04	0.437	–0.04	0.02	0.105
Crash count	0.02	0.03	0.480	0.05	0.03	0.242	0.02	0.03	0.480	0.04	0.03	0.253
Hard braking rates	0.04	0.05	0.947	0.00	0.02	0.967	0.03	0.05	0.947	–0.01	0.02	0.967
Hard cornering rate	0.03	0.05	0.766	0.01	0.04	0.866	0.01	0.05	0.766	0.00	0.04	0.982

Standardized beta coefficients (β), standard errors (SE), and FDR-adjusted *P* values for the main effects of WMH (total and regional), as well as the interaction effects between time and WMH on various driving performance metrics are shown. * Significant associations at *P* <0.05. Abbreviations: WMH, white matter hyperintensities.

© 2026 Parihar M et al. *JAMA Network Open.*

eTable 3. Demographic, Clinical, and WMH Characteristics by Cognitive Status During Follow-Up

	**Normal Cognition (*N*=182)**	**Cognitive Decline (*N*=38)**	***P* value**
Age (years)	72.30 (5.00) [63.00 - 92.00]	75.55 (4.18) [68.00 - 86.00]	<0.001***
Education (years)	16.62 (2.23) [12.00 - 24.00]	16.66 (2.13) [12.00 - 20.00]	0.8
Female, N (%)	85 (47%)	16 (42%)	0.6
Race, Caucasian, N (%)	161 (88%)	33 (87%)	0.8
Medications, Anti-Hypertensive, N (%)	118 (70%)	17 (47%)	0.005**
Area Deprivation Index	43.27 (21.94) [2.00 - 100.00]	45.16 (24.95) [4.00 - 100.00]	0.8
Total WMH volume (cm^3)	18.49 (12.55) [2.39 - 70.99]	23.07 (12.92) [6.76 - 56.79]	0.008**
Juxtacortical WMH (cm^3)	1.94 (2.81) [0.00 - 17.54]	2.15 (2.56) [0.15 - 11.73]	0.2
Frontal WMH (cm^3)	1.87 (2.44) [0.00 - 14.98]	2.52 (2.77) [0.29 - 12.38]	0.030*
Periventricular WMH (cm^3)	3.76 (1.49) [0.82 - 7.34]	4.39 (1.54) [0.67 - 7.10]	0.015*
Parietal WMH (cm^3)	4.80 (2.41) [0.46 - 10.41]	5.64 (2.32) [1.63 - 10.77]	0.039*
Posterior WMH (cm^3)	6.12 (4.86) [0.72 - 25.94]	8.37 (5.39) [2.10 - 24.06]	0.003**
10-year Framingham Stroke Risk Profile	6.43 (1.31) [3.23 - 10.28]	6.35 (1.47) [3.69 - 8.75]	>0.9
Follow-up duration (years)	5.63 (1.76) [0.60 - 9.70]	5.41 (1.75) [1.30 - 9.70]	0.2
Driving Cessation, N	30 (19.7%)	15(44.1%)	0.006**

Mean (SD) [Min – Max]; Abbreviations: WMH, white matter hyperintensities

© 2026 Parihar M et al. *JAMA Network Open.*

eFigure. Longitudinal Change in Adverse Driving Events by Cognitive Status. Average annual rates of adverse driving events are shown for participants who remained cognitive normal (red) and those who developed cognitive impairment (blue) over 10 years of follow-up. Adverse driving outcomes include (A) hard braking events, (B) crashes, (C) proportion of time spent overspeeding, and (D) hard cornering events. Bars reflect mean values with error bars representing standard errors. Participants who developed cognitive impairment showed marked increases in driving errors (hard braking and crash rates), despite reduced risky driving behaviors (overspeeding and hard cornering events) in later years, compared to those who remained cognitively normal.

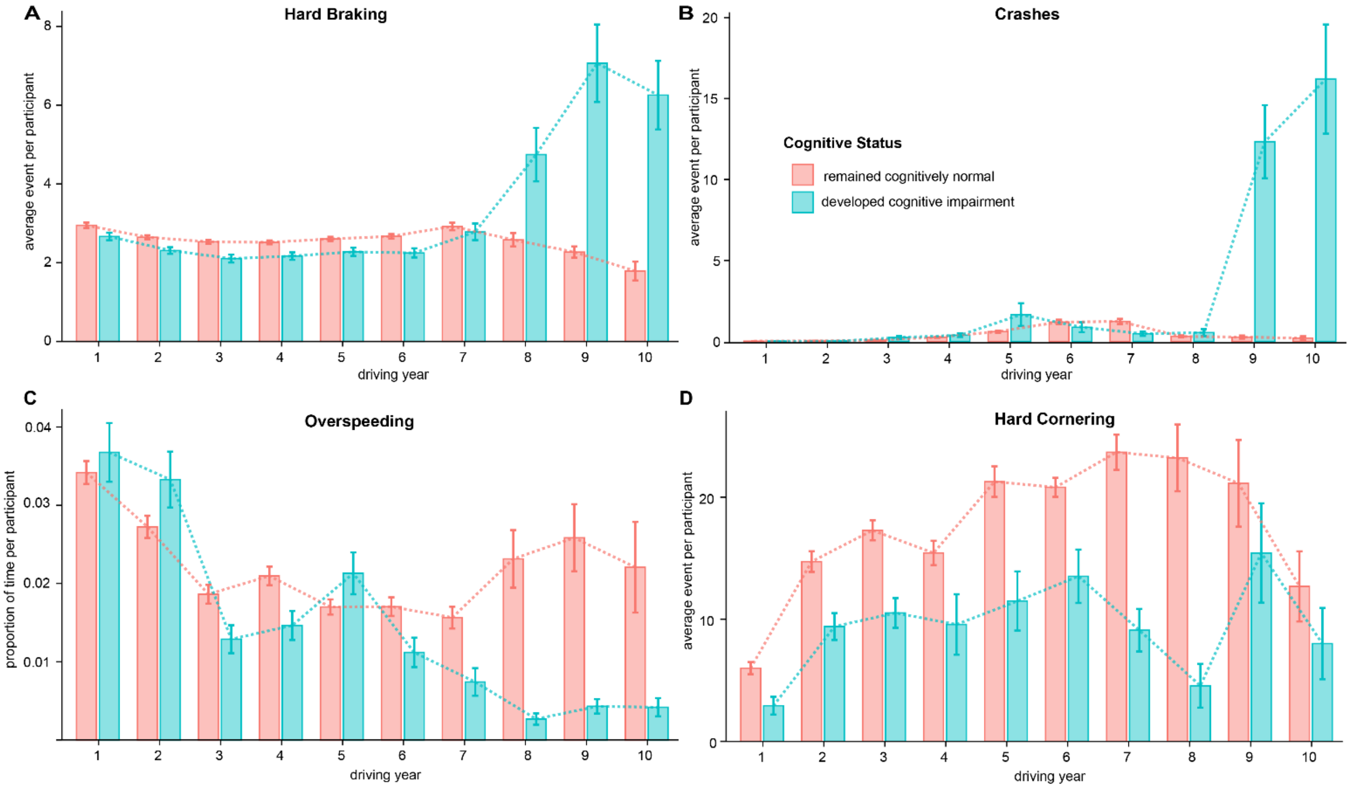

© 2026 Parihar M et al. *JAMA Network Open.*

eTable 4. Main Effects of Total and Regional WMH Burden, Time, and Cognitive Impairment on Driving Outcomes

	**Total WMH**			**Time*Total WMH**			**Time*Total WMH*CI**		
	β	SE	*P* value	β	SE	*P* value	β	SE	*P* value
Crash count	0.00	0.03	0.985	0.01	0.04	0.867	**0.23**	**0.11**	**0.032***
Hard braking rates	0.02	0.05	0.708	–0.01	0.02	0.655	**0.16**	**0.06**	**0.005***
Hard cornering rate	0.04	0.05	0.461	0.05	0.04	0.232	–0.04	0.12	0.754
	**Posterior WMH**			**Time*Posterior WMH**			**Time*Posterior WMH*CI**		
	β	SE	*P* value	β	SE	*P* value	β	SE	*P* value
Crash count	0.00	0.03	0.996	0.01	0.04	0.881	**0.24**	**0.11**	**0.060ᶲ**
Hard braking rates	0.00	0.05	0.942	–0.01	0.02	0.776	**0.15**	**0.06**	**0.023***
Hard cornering rate	0.04	0.05	0.826	0.05	0.04	0.491	–0.03	0.12	0.825
	**Parietal WMH**			**Time*Parietal WMH**			**Time*Parietal WMH*CI**		
	β	SE	*P* value	β	SE	*P* value	β	SE	*P* value
Crash count	0.00	0.03	0.996	0.01	0.04	0.881	0.18	0.10	0.088
Hard braking rates	–0.02	0.05	0.880	0.01	0.02	0.776	0.09	0.05	0.092
Hard cornering rate	0.05	0.05	0.826	0.04	0.04	0.491	–0.05	0.11	0.825
	**Periventricular WMH**			**Time*Periventricular WMH**			**Time*Periventricular WMH*CI**		
	β	SE	*P* value	β	SE	*P* value	β	SE	*P* value
Crash count	0.00	0.03	0.996	–0.01	0.04	0.881	**0.25**	**0.09**	**0.033***
Hard braking rates	0.02	0.05	0.880	–0.02	0.02	0.756	**0.11**	**0.05**	**0.036***
Hard cornering rate	0.01	0.05	0.826	0.08	0.04	0.357	–0.03	0.10	0.825
	**Frontal WMH**			**Time*Frontal WMH**			**Time*Frontal WMH*CI**		
	β	SE	*P* value	β	SE	*P* value	β	SE	*P* value
Crash count	0.01	0.03	0.996	0.02	0.04	0.881	0.15	0.11	0.146
Hard braking rates	0.03	0.05	0.880	–0.01	0.02	0.756	**0.15**	**0.06**	**0.023***
Hard cornering rate	0.02	0.05	0.826	0.02	0.04	0.717	–0.03	0.11	0.825
	**Juxtacortical WMH**			**Time*Juxtacortical WMH**			**Time*Juxtacortical WMH*CI**		
	β	SE	*P* value	β	SE	*P* value	β	SE	*P* value
Crash count	0.01	0.03	0.996	0.01	0.04	0.881	0.21	0.11	0.088
Hard braking rates	0.02	0.05	0.880	–0.02	0.02	0.756	0.10	0.06	0.092
Hard cornering rate	0.01	0.05	0.826	0.01	0.04	0.830	–0.06	0.12	0.825

Abbreviations: β, mixed model estimates; SE, standard errors; CI, cognitive impairment; WMH, white matter hyperintensities. * Significant three-way interactions (FDR-adjusted *P*<0.05) indicate longitudinal association between total or regional WMH burden and driving outcomes differs by cognitive status. ᶲ Additional associations meeting unadjusted significance thresholds (*P*<0.05)

© 2026 Parihar M et al. *JAMA Network Open.*

eTable 5. Demographic, Clinical, and WMH Characteristics by MRI Follow-up Status

	**Baseline MRI Only (*N*=107)**	**With Follow-Up MRI (*N*=113)**	***P* value**
Age at baseline MRI (years)	73.51 (5.44) [63.00 - 92.00]	72.24 (4.52) [64.00 - 86.00]	0.07
Interval between MRI scans (years)	*na*	4.03 (1.25) [1.41 - 7.31]	*na*
Education (years)	16.71 (2.15) [12.00 - 24.00]	16.55 (2.26) [12.00 - 20.00]	>0.9
Female, N (%)	49 (46%)	52 (46%)	>0.9
Race, Caucasian, N (%)	91 (85%)	103 (91%)	0.2
Cognitive Impairment, N (%)	20 (19%)	18 (16%)	0.6
Medications, Anti-Hypertensive, N (%)	74 (73%)	61 (60%)	0.06
Area Deprivation Index	45.41 (23.95) [3.00 - 100.00]	41.88 (20.87) [2.00 - 100.00]	0.4
Baseline MRI			
Total WMH volume (cm3)	21.96 (14.38) [2.39 - 70.99]	16.75 (10.32) [3.50 - 58.78]	0.005**
Juxtacortical WMH (cm3)	2.40 (3.11) [0.00 - 17.54]	1.58 (2.32) [0.02 - 12.01]	0.020*
Frontal WMH (cm3)	2.44 (2.93) [0.00 - 14.98]	1.55 (1.92) [0.10 - 12.38]	0.011*
Periventricular WMH (cm3)	4.16 (1.55) [1.21 - 7.14]	3.58 (1.42) [0.67 - 7.34]	0.007**
Parietal WMH (cm3)	5.34 (2.48) [0.46 - 10.77]	4.58 (2.29) [0.61 - 10.33]	0.023*
Posterior WMH (cm3)	7.62 (5.76) [0.72 - 24.56]	5.45 (3.94) [0.84 - 25.94]	0.006**
Follow-up MRI			
Total WMH volume (cm3)	*na*	18.63 (10.87) [3.24 - 63.91]	*na*
Juxtacortical WMH (cm3)	*na*	1.94 (2.47) [0.02 - 13.98]	*na*
Frontal WMH (cm3)	*na*	1.90 (2.13) [0.13 - 10.63]	*na*
Periventricular WMH (cm3)	*na*	3.87 (1.39) [0.14 - 7.50]	*na*
Parietal WMH (cm3)	*na*	4.69 (2.47) [0.37 - 10.82]	*na*
Posterior WMH (cm3)	*na*	6.23 (4.31) [1.01 - 28.11]	*na*
10-year Framingham Stroke Risk Profile	6.44 (1.44) [3.23 - 10.28]	6.39 (1.23) [3.94 - 8.86]	0.8

Mean (SD) [Min – Max]; Abbreviations: WMH, white matter hyperintensities; *na*, not applicable

© 2026 Parihar M et al. *JAMA Network Open.*

eTable 6. Main Effects of Posterior WMH Burden, Growth, and Cognitive Impairment on Driving Safety Outcomes, With and Without AD Pathology Adjustment. This table summarizes longitudinal model results examining associations of posterior WMH growth (Model 1); posterior WMH burden and cognitive impairment (Model 2); posterior WMH growth and cognitive impairment (Model 3), including their interactions with time, on driving safety outcomes. Results are shown for both baseline models and models adjusted for AD pathology (amyloid Centiloid and tau PET SUVR).

		**Primary Model**						**AD pathology adjustment**					
**Model 1**		**Posterior WMH growth**			**Time*Posterior WMH growth**			**Posterior WMH growth**			**Time*Posterior WMH growth**		
		β	SE	*P* value	*β*	SE	*P* value	*β*	SE	*P* value	*β*	SE	*P* value
	Crash count	**0.43**	**0.15**	**0.020***	0.34	0.13	0.051	**0.65**	**0.23**	**0.034***	**0.57**	**0.21**	**0.042***
	Hard braking rates	0.07	0.06	0.573	–0.02	0.02	0.534	0.04	0.08	0.801	0.00	0.02	0.994
**Model 2**		**Time*Posterior WMH**			**Time*Posterior WMH*CI**			**Time*Posterior WMH**			**Time*Posterior WMH*CI**		
		β	SE	*P* value	*β*	SE	*P* value	*β*	SE	*P* value	*β*	SE	*P* value
	Crash count	0.01	0.04	0.881	0.24	0.11	0.060	0.00	0.06	0.977	**0.42**	**0.18**	**0.032***
	Hard braking rates	–0.01	0.02	0.776	**0.15**	**0.06**	**0.023***	-0.02	0.02	0.876	**0.17**	**0.07**	**0.029***
**Model 3**		**Time*Posterior WMH growth**			**Time*Posterior WMH growth*CI**			**Time*Posterior WMH growth**			**Time*Posterior WMH growth*CI**		
		β	SE	*P* value	*β*	SE	*P* value	*β*	SE	*P* value	*β*	SE	*P* value
	Crash count	-0.03	0.12	0.897	**1.71**	**0.27**	**3e–08***	0.00	0.18	0.993	**2.84**	**0.42**	**3e-08***
	Hard braking rates	–0.03	0.03	0.611	0.07	0.06	0.396	-0.02	0.02	0.493	**0.14**	**0.07**	**0.042***

Abbreviations: β, mixed model estimates; SE, standard errors; CI, cognitive impairment; WMH, white matter hyperintensities; AD, Alzheimer's disease *Significance levels at FDR-adjusted P <0.05

© 2026 Parihar M et al. *JAMA Network Open.*

eTable 7. Demographic, Clinical, and WMH Characteristics by Antihypertensive Therapy Status

	**No antihypertensive therapy (*N*=69)**	**On antihypertensive therapy (*N*=135)**	***P* value**
Age (years)	72.41 (4.77) [63.00 - 86.00]	73.16 (5.26) [65.00 - 92.00]	0.5
Education (years)	16.83 (2.30) [12.00 - 20.00]	16.45 (2.21) [12.00 - 24.00]	0.12
Female, N (%)	35 (51%)	60 (44%)	0.4
Race, Caucasian, N (%)	66 (96%)	114 (84%)	0.019*
Cognitive Impairment, N (%)	19 (28%)	17 (13%)	0.008**
Area Deprivation Index	38.10 (19.70) [2.00 - 78.00]	46.16 (23.58) [4.00 - 100.00]	0.039*
Total WMH volume (cm^3)	17.94 (11.90) [3.50 - 58.78]	20.35 (13.39) [2.39 - 70.99]	0.2
Juxtacortical WMH (cm^3)	1.75 (2.46) [0.02 - 11.93]	2.16 (2.99) [0.00 - 17.54]	0.3
Frontal WMH (cm^3)	1.78 (2.35) [0.10 - 12.38]	2.18 (2.67) [0.00 - 14.98]	0.3
Periventricular WMH (cm^3)	3.75 (1.53) [0.93 - 7.34]	3.92 (1.53) [0.67 - 7.14]	0.5
Parietal WMH (cm^3)	4.66 (2.26) [0.48 - 10.33]	5.17 (2.52) [0.46 - 10.77]	0.3
Posterior WMH (cm^3)	6.00 (4.70) [0.91 - 25.94]	6.92 (5.28) [0.72 - 24.56]	0.3
10-year Framingham Stroke Risk	5.89 (1.47) [3.23 - 8.41]	6.68 (1.20) [4.31 - 10.28]	<0.001***
Antihypertensive class, N (%)			
ACE inhibitors	*na*	55 (41%)	*na*
Angiotensin II Receptor Blockers	*na*	48 (36%)	*na*
Beta-blockers	*na*	56 (41%)	*na*
Calcium Channel Blockers	*na*	60 (44%)	*na*
Diuretics	*na*	55 (41%)	*na*
Blood Pressure Controlled, N (%)	24 (35%)	21 (16%)	0.002**
Follow-up duration (years)	5.59 (1.81) [1.10 - 9.50]	5.61 (1.77) [1.00 - 9.70]	>0.9

Mean (SD) [Min – Max]; Abbreviations: WMH, white matter hyperintensities; ACE, angiotensin-converting enzyme; *na*, not applicable

© 2026 Parihar M et al. *JAMA Network Open.*

eTable 8. Longitudinal Association Between Periventricular WMH and Driving Behaviors Varies by HTN Treatment and BP Control Status. G0: no HTN medications, poor BP control; G1: no HTN medications, good BP control; G2: on HTN medications, poor BP control; G3: on HTN medications, good BP control.

		**G0: Time*Periventricular WMH**			**G1: Time*Periventricular WMH**			**G2: Time*Periventricular WMH**			**G3: Time*Periventricular WMH**		
		β	SE	*P* value	*β*	SE	*P* value	*β*	SE	*P* value	*β*	SE	*P* value
**Any**	Crash count	–0.01	0.08	0.900	0.01	0.13	0.934	0.13	0.09	0.188	–0.09	0.13	0.959
	Hard cornering rate	**0.27**	**0.08**	**0.004***	–0.27	0.14	0.247	**–0.26**	**0.10**	**0.041***	–0.29	0.14	0.162
**ACE inhibitors**	Crash count	–0.01	0.09	0.908	0.01	0.15	0.935	0.20	0.13	0.300	0.01	0.19	0.991
	Hard cornering rate	**0.27**	**0.09**	**0.011***	–0.27	0.15	0.375	–0.31	0.13	0.081	–0.39	0.19	0.185
**ARB**	Crash count	–0.01	0.09	0.916	0.01	0.16	0.935	0.23	0.13	0.142	–0.32	0.32	0.799
	Hard cornering rate	**0.27**	**0.10**	**0.045***	–0.28	0.18	0.574	–0.27	0.14	0.854	–0.27	0.36	0.791
**CCB**	Crash count	–0.01	0.08	0.896	0.01	0.14	0.926	0.25	0.12	0.093	0.02	0.27	0.956
	Hard cornering rate	**0.27**	**0.08**	**0.009***	–0.27	0.15	0.336	–0.28	0.12	0.119	–0.25	0.28	0.786
**Beta Blockers**	Crash count	–0.01	0.09	0.917	0.01	0.16	0.937	0.17	0.13	0.394	–0.13	0.19	0.937
	Hard cornering rate	**0.27**	**0.10**	**0.038***	–0.28	0.17	0.551	–0.22	0.14	0.549	–0.29	0.21	0.791
**Diuretics**	Crash count	–0.011	0.04	0.824	0.01	0.08	0.856	0.02	0.06	0.784	0.00	0.19	0.991
	Hard cornering rate	**0.27**	**0.09**	**0.011***	–0.27	0.15	0.381	–0.25	0.13	0.254	–0.18	0.37	0.854

Abbreviations: β, mixed model estimates; SE, standard errors; HTN, hypertension; WMH, white matter hyperintensities; ACE, angiotensin-converting enzyme; ARB, angiotensin II receptor blocker; CCB, calcium channel blocker. * Significant three-way interactions (FDR-adjusted $P < 0.05$) indicate longitudinal association between periventricular WMH burden and driving outcomes differs by hypertension treatment and BP control status.

© 2026 Parihar M et al. *JAMA Network Open.*
