# Supplementary material for: White Matter Hyperintensity Burden and Decline in Driving Performance Among Older Adults
Source: JAMA Netw Open. 2026 Jan 29;9(1):e2554501. doi: 10.1001/jamanetworkopen.2025.54501 (PMC12856682; doi:10.1001/jamanetworkopen.2025.54501)
Supplement: Supplement 2. — Nonauthor Collaborators. Driving Real-World In-Vehicle Evaluation System (DRIVES) Project [file jamanetwopen-e2554501-s002.pdf]

Supplemental Online Content: Nonauthor Collaborators
*First name, last name, and suffix (if applicable) are required and will appear in PubMed.

*Group Name(s): The Driving Real-World In-Vehicle Evaluation System (DRIVES) Project							
***First Name and Middle Initial(s)**	***Last Name**	***Suffix (eg, Jr, III)**	Academic Degrees	Institution	Location (city, state/province, country)	Role or Contribution, eg, chair, principal investigator	Group (if more than 1 Group listed in the byline) and/or Subgroup (eg, Steering Committee)
Kaylin	Taylor		BA, MS	Washington University School of Medicine	St. Louis, Missouri, USA	Clinical Research Coordinator	The DRIVES Project
Maeve	Intagliata		BS, MS	Washington University School of Medicine	St. Louis, Missouri, USA	Clinical Research Coordinator	The DRIVES Project
Nikitha	Damera		BA	Washington University School of Medicine	St. Louis, Missouri, USA	Clinical Research Coordinator	The DRIVES Project
Carson	Woodfin		BS	Washington University School of Medicine	St. Louis, Missouri, USA	Clinical Research Coordinator	The DRIVES Project
Ann M.	Johnson		HS	Washington University School of Medicine	St. Louis, Missouri, USA	Clinical Research Coordinator	The DRIVES Project
Matthew	Blake		BA, MS	Washington University School of Medicine	St. Louis, Missouri, USA	Bioinformaticist	The DRIVES Project
Chen	Chen		BA, MSW, MPH	Washington University School of Medicine	St. Louis, Missouri, USA	Statistical Data Analyst	The DRIVES Project
