# Supplementary material for: White Matter Hyperintensity Burden and Decline in Driving Performance Among Older Adults
Source: JAMA Netw Open. 2026 Jan 29;9(1):e2554501. doi: 10.1001/jamanetworkopen.2025.54501 (PMC12856682; doi:10.1001/jamanetworkopen.2025.54501)
Supplement: Supplement 3. — Data Sharing Statement [file jamanetwopen-e2554501-s003.pdf]

Data Sharing Statement

Parihar. White Matter Hyperintensity Burden and Decline in Driving Performance Among Older Adults. *JAMA Netw Open*. Published January 27, 2026.
doi:10.1001/jamanetworkopen.2025.54501

Data
Data available: No

Additional Information
Explanation for why data not available: The data underlying this study are not publicly available due to privacy concerns, as latitude and longitude coordinates could be used to identify participants or their residences. Data may be available from the corresponding author upon reasonable request and with appropriate protections.
